# Realist Evaluation of the “Abiye” Safe Motherhood Initiative in Nigeria: Unveiling the Black-Box of Program Implementation and Health System Strengthening

**DOI:** 10.3389/frhs.2022.779130

**Published:** 2022-05-10

**Authors:** Olawale J. Oladimeji, Adesegun O. Fatusi

**Affiliations:** ^1^Academy for Health Development, Ile-Ife, Nigeria; ^2^Office of the Vice-Chancellor, University of Medical Sciences, Ondo, Nigeria; ^3^Department of Community Health, Faculty of Clinical Sciences, College of Health Sciences, Obafemi Awolowo University, Ile-Ife, Nigeria

**Keywords:** “Abiye”, safe motherhood initiative, context, mechanism, outcome, health system strengthening, realist evaluation

## Abstract

**Introduction:**

Realist evaluation studies have spanned different aspects of medicine, especially in the field of public health. However, very few of these studies explicitly detailed how program implementation triggered outcomes that could strengthen understanding of its effect on Health System Strengthening in specific settings. In low- and middle-income countries, like Nigeria, there is a paucity of realist evaluation studies, despite the implementation of multiple intervention programs and projects in these countries. This article is aimed at unveiling the black-box of program implementation and Health System Strengthening of the “Abiye” Safe Motherhood Program in Ondo State, Nigeria. Specifically, it identified the role of contextual factors in the “Abiye” program in Ondo State, determined the mechanisms that facilitated or constrained outcomes of the “Abiye” program, and developed a Context Mechanism Outcome (CMO) Configuration from which a Middle Range Theory (MRT) can be framed.

**Methodology:**

This was qualitative research structured along with the realist domains (Context, Mechanism, and Outcome). The Initial Program Theory was validated by the qualitative study, after which a new MRT was developed. The study population comprised key stakeholders, secondary stakeholders, and primary stakeholders in the Abiye safe motherhood program. Data was collected through 10 key informant interviews, 28 in-depth interviews, and six focus group discussions sessions. Thematic analysis was used to analyze all the qualitative data collected, and seven themes with 19 subthemes emerged in the study.

**Results:**

We identified 13 contextual factors under five principal areas, with most of the factors playing enabling roles, some playing inhibitory roles, while very few played both roles. We elicited eight mechanisms, and some of these facilitated the outcomes, while some constrained the outcomes of the program. Health system strengthening was a key feature of the outcome of the program. We developed a middle-range theory based on the 6 CMO configurations we elicited from the study.

**Conclusion and Policy Implications:**

Realist evaluation is an iterative process that looks beyond the surface to generate evidence. By applying the realist approach, we generated pieces of evidence that can be adapted for policymaking in public health interventions in LMIC.

## Introduction

Realist evaluation is a form of theory-based evaluation and is unique because of the assumption that nothing works everywhere for everyone (1). It uses a social science paradigm (2) which recognizes the multiple intertwined factors in society, making it appropriate for evaluating programs and policies with complex social interaction. It focuses on what works for whom, why, how and under what circumstances (3). Context, mechanism, and outcome are the main domains in which it is implemented. Context is the setting, either internal or external, in which it is being implemented (4). The contextual factors have been classified into situational, structural, socio-cultural, or international using a simplified framework (5). Mechanisms have been described as reasoning or the process (that may be hidden) by which actors trigger an outcome (6). In realism, outcomes could be multiple, based on the mechanism activated and in a specific context. The context, mechanism, and outcome form a configuration; hereafter, the CMO configuration (1, 6).

Realist evaluation studies have spanned different aspects of medicine, especially in the field of public health. However, very few of these studies explicitly detailed how outcomes were triggered by program implementation, which led to health system strengthening in specific settings (6–8). In low and middle-income countries, like Nigeria, there is a paucity of realist evaluation studies with evidence of health system strengthening (9, 10), despite the implementation of multiple intervention programs and projects in the health systems of these countries. Consequently, the opportunities to derive critical lessons for policymaking from previous programs are lost, and replication, as well as scaling up of programs, becomes challenging. Previous realist evaluation publications from Africa have focused on the domains of the realist approach (11, 12) while related health system strengthening by such public health programs needs further evaluation (13).

The “*Abiye*” (Safe Motherhood in the Yoruba language) program was initiated in 2009 and stopped in early 2017 in Ondo State, Nigeria to address the challenges of poor maternal and child health outcomes in the state. The initiative sought to address the four levels of delay (14) associated with maternal mortality—(i) delay in seeking quality care, (ii) delay in reaching care, (iii) delay in receiving care, and (iv) delay in being referred for appropriate care. The “Abiye” program was initiated in 2009 and implemented in Ifedore LGA as a pilot, and then it was later extended to all the other 17 local government areas in the state by 2012 (15, 16). The program was implemented along demand and supply components to make definitive changes in the health system (15). The demand component focuses on increasing utilization at the health facilities by making all the services free, assigning each pregnant woman to a community health extension worker (Health Ranger), and strengthening referrals to the appropriate facilities. The supply component, on the other hand, focuses on renovating and equipping the health facilities with drugs and life-saving materials and supplying adequate human resources for health. Previous evaluative studies on the “Abiye” program have focused on different aspects of the program in specific parts of the state using the logic model concept but did not elicit the multi-dimensional interactions associated with the program (17–19).

This article is aimed at unveiling the black-box of program implementation and Health System Strengthening of the “Abiye” Safe Motherhood Program in Ondo State, Nigeria. Specifically, it identified the role of contextual factors in the “Abiye” program in Ondo State, determined the mechanisms that facilitated or constrained outcomes of the “Abiye” program, and developed a CMO Configuration from which a Middle-Range Theory (MRT) was framed.

## Methods

### Realist Approach

Three steps are involved in the realist evaluation approach (1, 6, 7): (i) development of the Initial Program Theory (IPT); (ii) testing and validation of the theory; and (iii) refining the theory from the CMO Configuration to develop the middle-range theory.

#### Initial Program Theory Development

**Figure 1:**
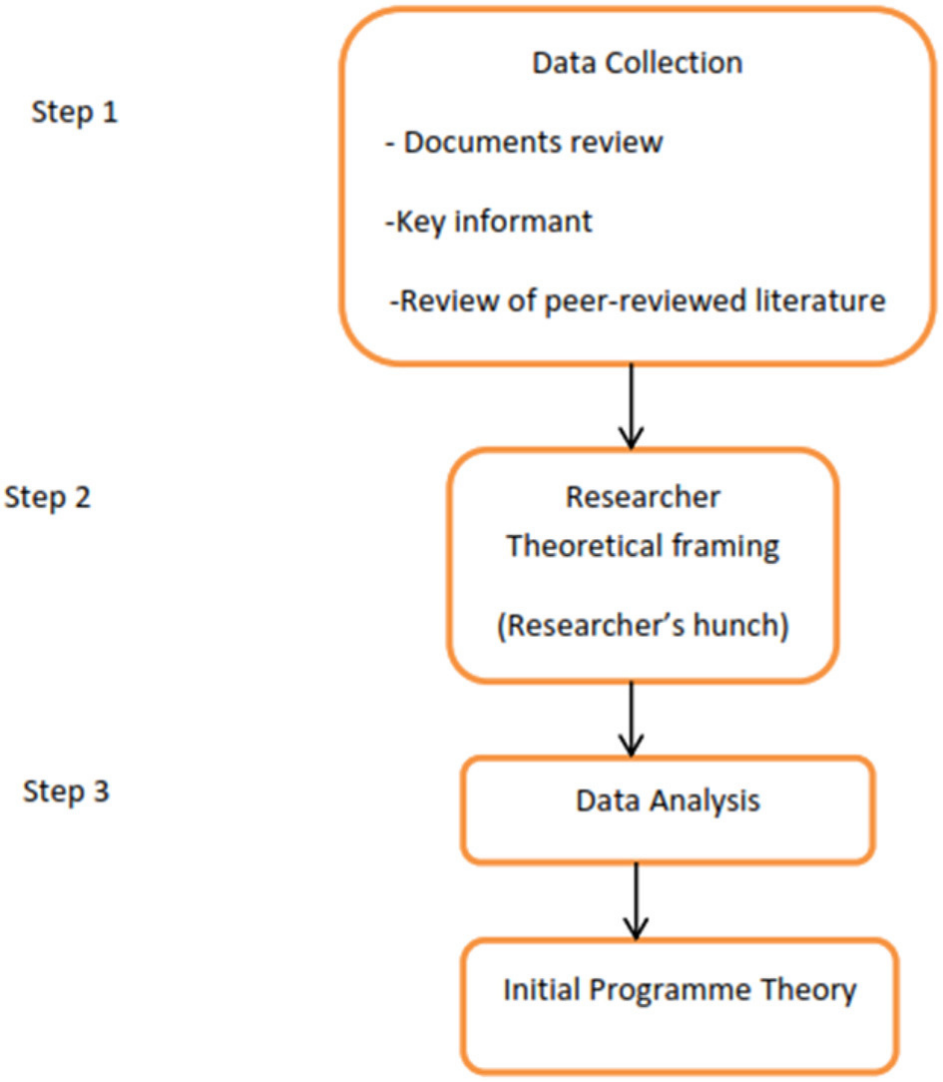
Steps for developing the Initial Program Theory for the “Abiye” safe motherhood program.

**Figure 2:**
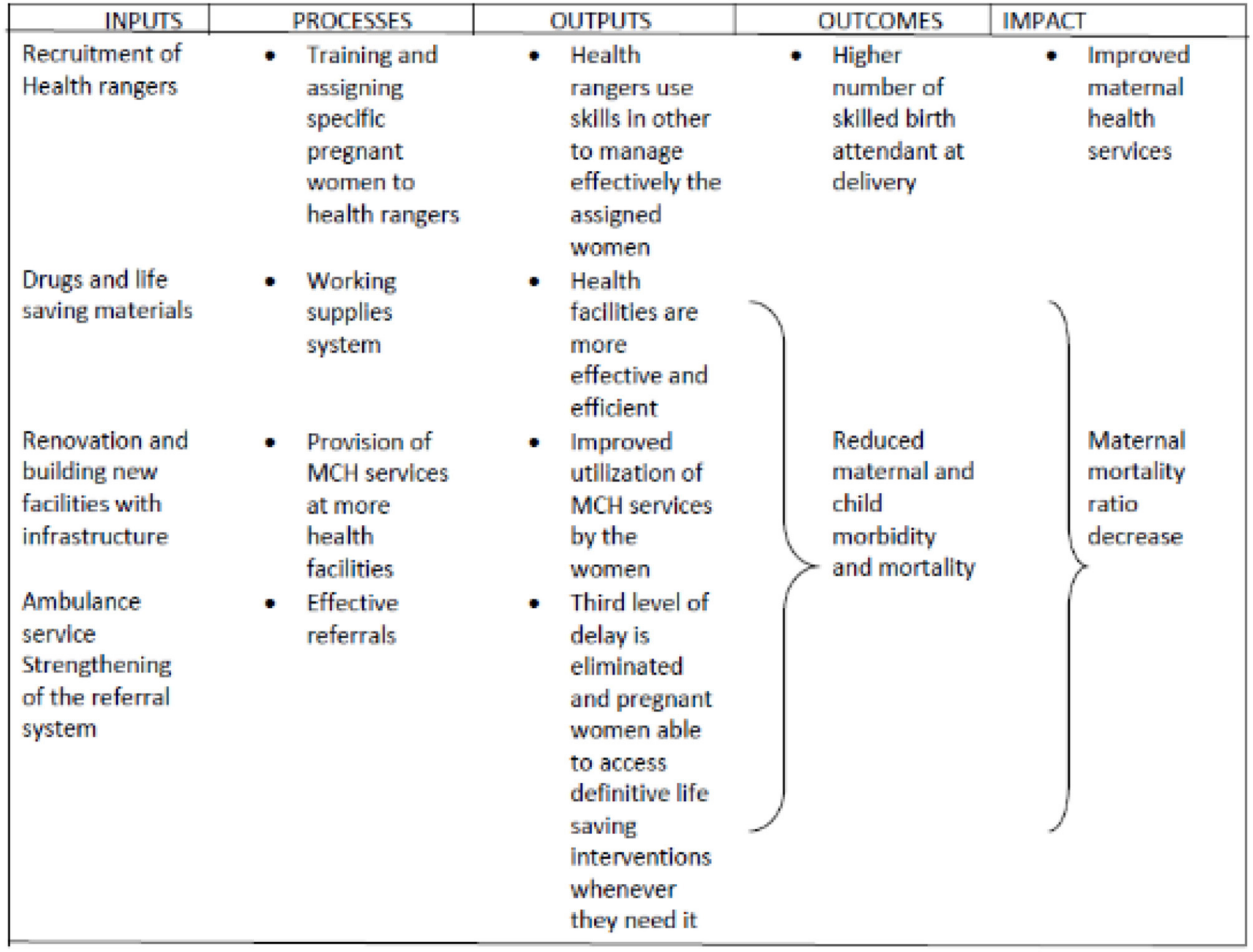
The logic model of the “Abiye” safe motherhood program.

Two initial program theories (demand and supply components) were developed. The initial program theories were framed around the core program strategies, which are the demand and supply components of the program. These theories were developed by adapting methods previously published in realist evaluation IPT development studies (20–24), and were broken down into three steps (Figure 1). The summary of the data sources for the development of the initial program theory is in Table 1. A logic model was also developed from the data sources to reflect the logic framework for the implementation of the program (Figure 2).

**Table 1:** Summary of the data sources for the development of the Initial Program Theory.

**Documents reviewed**	**Key informant's interviewed**	**Peer-reviewed literature reviewed**
Evaluation of Abiye safe motherhood program on Ondo. Reports. 2012 Speech on the introduction of “Abiye” program in Ondo State. 2009 Ondo State government 2015 “Abiye” safe motherhood. Ondo State Ministry of Health. 2015 Ondo State Primary Health Care Development Board 2013–2017. Reports	3 policymakers in Ondo State government 3 program developers in Ondo State 6 program implementers in Ondo State	Safe Motherhood Initiative. 2007 The Single Best Intervention: Thirty Years of Safe Motherhood Cost-effective safe motherhood interventions in low-income countries: a review Policy Trends in Advancing Safe Motherhood Safe Motherhood: a brief history of the global movement Thirty Years of the Safe Motherhood Initiative: Celebrating Progress and Charting the Way Forward

##### Demand Component—Initial Program Theory

“Community engagement strengthened referral, and an effective ambulance system without delay in reaching or receiving quality health care whenever or wherever it is needed would build trust, commitment, and participation of the community women (and their households) in the health system and translate into improved access and utilization of the health facilities, thereby improving maternal health and reducing maternal and infant mortality in Ondo State.”

##### Supply Component—Initial Program Theory

The availability of highly motivated, skilled healthcare personnel with an appropriate service delivery environment and functional equipment that was previously unavailable would result in quality maternal care in Ondo State, thereby reducing maternal and child mortality. See Table 2 for the matrix of the context, mechanism, and outcome for the demand and supply components of the IPT.

**Table 2:** Matrix of the context, mechanism and outcome for demand and supply component of the IPT.

**Context**	**Mechanism**	**Outcome**
**Demand component**		
Community engagement Strengthened referral Effective ambulance system	Community participation Trust Commitment	Improved utilization Improved access Reduced maternal mortality
**Supply component**		
Skilled health personnel Functional equipment	Motivated Appropriate service delivery environment	Quality maternal care Reduced maternal mortality

#### Validation by the Qualitative Method of the Study

The initial program theories developed were tested by the qualitative method which allowed the exploration of the context, mechanisms, and outcome of the study using Key Informant Interviews, In-Depth Interviews, and Focus Group Discussion sessions applied during the study. This method tested both the demand and supply components of the IPT along with the developed Logic Model. This was done by exploring the items listed under the IPT domains and the constructs in the Logic Model.

#### Refined Program Theory

The initially developed program theory was refined and remodeled after validation by the qualitative method applied to generate a CMO Configuration from which a new theory, the MRT, was developed.

### Methodology

This is qualitative research that was conducted in Ondo State, South-West Nigeria. The state has three senatorial districts and eighteen local government areas (six in each senatorial district). The study population was comprised of (i) key stakeholders, who were policymakers, such as the “Immediate Past Governor” of the State, the Immediate Past Commissioner of Health in the State, Directors in the Ministry of Health, and relevant departments who were present during the implementation of the program. They were included because they were critical in the agenda-setting, design, decision-making, and implementation of the “Abiye” program in the state. (ii) Secondary stakeholders included service delivery personnel, such as Maternity In-Charges, Health Rangers (community health extension workers specifically engaged for the “Abiye” program and assigned 25 pregnant women in the community to each of them to follow-up until delivery), nurses, and midwives who were directly involved in the state's implementation of the “Abiye” program. They were included because of their experiences and perspectives on the role of contextual factors and mechanisms in the implementation process and the outcomes that occurred. (iii) primary stakeholders, who were the program beneficiaries, particularly mothers who delivered during the program. Mothers who delivered before and during the program period, as well as older women in the community, were also included. The mothers who delivered during the program had experiences of how contextual factors and mechanisms affected the outcomes, while the mothers who gave birth before and during the program had broad perspectives on contextual factors and mechanisms that were different during their two delivery experiences. The older community women had experiences and perspectives regarding the pre-“Abiye” program period as well as witnessed the unfolding and implementation of the “Abiye” program and they provided insight into these periods.

The qualitative data collection methods that were used in this study were focus group discussions (FGD) in which the primary stakeholders participated, in-depth interviews (IDI) in which the secondary stakeholders participated, and key informant interviews (KII) in which the key stakeholders were the interviewees. The participants for the FGD and IDI were selected through a probable sample procedure—multistage sampling with the senatorial districts, Local Governments Areas (LGAs), wards as the sampling frames, and then participants were selected purposively from the wards. Furthermore, three LGAs (one from each senatorial district in the state) were selected for the study and six wards (two from each selected local government) were selected for the study. The interviewees for the key informant interviews were purposely selected from the eligible directors and policymakers.

The study instruments were the FGD guide, the In-depth interview guide, and the KII guide, which was developed based on the contextual factors that influenced the agenda-setting, design, decision-making, and implementation of the program and on mechanisms that facilitated or constrained outcomes of the program.

#### Data Collection Methods

During the study, 10 KII sessions were held. Key informant interviews were held with the immediate past “governor” of Ondo State, the immediate past commissioner of health of Ondo State, five directors from relevant ministries, departments, and agencies in Ondo State, and the local government primary health care coordinators in the selected LGAs. Data collection for the key stakeholders was stopped when data saturation was reached, that is when a further interview with additional key stakeholders did not yield any new information for the study (25). The KII sessions were conducted in the English language and tape-recorded after seeking permission from the respondents.

During the study, 28 IDI sessions were held. IDIs were held with six health rangers (one from each of the six selected wards) and six facility health workers (one from each of the selected wards). Also, in-depth interviews were held with 15 women (five in each LGA) who gave birth before and during the “Abiye” program. This was to help elicit the experiences and perspectives before and during the program, and this was in keeping with the logic of using a small sample in interview-based qualitative research (26). A traditional birth attendant who stopped practicing during the “Abiye” program was also interviewed to give further insight into the perspectives of non-implementing stakeholders. All the IDIs were tape-recorded after due consent was given by the interviewees. Some of the IDI were conducted in the English language but some were conducted in the Yoruba language for ease of communication, and then translated to English using a two-way translation approach. All translations were done by two postgraduate students in the Department of African Languages at Obafemi Awolowo University, Ile-Ife.

During the study, six focus group discussion sessions were held. In each of the selected LGAs, two FGD sessions were held, sessions for (i) mothers who had delivered in the “Abiye” program 2 years prior (2015–2016) to the end of the program and (ii) older women (age 60 years and above) in the community. Each FGD consisted of 6–12 participants. The pre-designed guide for FGD was translated into Yoruba, the predominant local language in the state, for ease of communication using a two-way translation approach and to ensure proper understanding. Each of the FGD sessions lasted about 30–45 min and was tape-recorded after seeking permission from the participants. The FGDs were conducted in the Yoruba language and the tape-recorded form was translated to English using a two-way translation approach. This was to ensure an accurate reflection of perspectives. All translations were done by two postgraduate students in the Department of African Languages at Obafemi Awolowo University, Ile-Ife (A student translated the tape-recorded Yoruba session to the English language while the other student translated it back to Yoruba, this was to ensure the information and perspectives from the interviews were retained). One of the authors (OJ) was the moderator in all the FGD sessions and made use of a trained note-taker who recorded key issues (through jottings and audio recording) during the sessions.

#### Outcome Measures

The measures for analyzing the role of contextual factors in the “Abiye” policy in Ondo State were derived from the perspective of key stakeholders, secondary stakeholders, and primary stakeholders on the role of contextual factors which were elicited during the KIIs, IDIs, and FGD sessions.

The measures for identifying the mechanisms that facilitated or constrained outcomes of the “Abiye” safe motherhood program were derived from the perspectives of the key stakeholders, health workers, mothers who delivered before and during the program, older women in the community, and TBA which were elicited from the KII, IDI, and FGD.

#### Data Analysis

Thematic analysis was carried out for all the data collected. Thematic analysis is a process of identifying patterns or themes within qualitative data (27). It is appropriate for realist approach studies because motivations, experiences, and meanings can be theorized from the themes (28). Deductive approach (29) was applied in this study. The themes were allowed to emerge freely from the collected data to reflect comprehensive perspectives of the “Abiye” Safe Motherhood program, then the deductive approach was also applied to map the three domains of realist evaluation (context, mechanisms, and outcome), which are central to the objectives of the study. The six-phase guideline of doing thematic analysis (27) was applied in the analysis of the qualitative data collected in this study. The application of this guideline was not linear but recursive, with movements along with the phases back and forth as needed.

##### Phase 1: Data Familiarization

The qualitative data collected in the Yoruba language from the FGDs and some of the IDIs were translated to the English language by a postgraduate student of the Department of African Studies of Obafemi Awolowo University and back to the Yoruba Language by another postgraduate student of the Department of African Studies using a two-way translation method to ensure the perspective and ideas in the collected data is not lost. All the qualitative data in English in this study were transcribed verbatim using a rigorous process that entailed listening multiple times to the recorded tapes thereby retaining the actual meaning in the verbal accounts which is essential in qualitative analysis (27). The transcription also ensured familiarization with the data and was critical in creating meaning for this thematic analysis. The transcribed data was read multiple times to further ensure adequate familiarization.

##### Phase 2: Initial Codes Generation

Initial codes were generated from the transcribed dataset of this study. Codes are the basic feature of data that can be interpreted in a meaningful way concerning the topic in focus (30). The coding was influenced by the data and the entire data set in the qualitative method was coded. Coding was done for the KII sessions separately, then for the IDI sessions and the FGD sessions. The coding was done initially manually (31), by systematically working through the transcript giving attention to the details in each extract and noting them, using pens and highlighters for potential themes or patterns. Further coding was done for the data set using Microsoft excel (32) to generate more codes from the data set and help identify themes. This process of coding manually and recoding using Microsoft excel was to ensure all possible codes from the data extracts contained in the data set were generated because of the different data collection methods used in the study. A long list of codes that formed a code book was generated from the entire data set which was summarized into a tabular form to reflect the deductive approach from which the thematic matrix was developed.

##### Phase 3: Searching for Themes

The long list of codes generated was sorted into potential themes according to the different patterns in the different data extracts. Essentially, the different codes generated from the KII, IDI, and FGD sessions were combined together, organized, and reorganized to form a different pattern to enable themes to emerge from the data set. A visual representation (33), a thematic map, was used to sort the different codes into themes. A thematic map is used to show relationships and links between codes, themes, and subthemes. Some of the codes did not fit appropriately into a theme and were classified into a theme called “miscellaneous theme” (27), which was further reviewed later. The themes identified in this phase of the analysis represented the initial theme (candidate themes) (33) of the dataset.

##### Phase 4: Reviewing Themes

The candidate themes initially identified in this study were further reviewed to refine and assess their uniqueness. Internal homogeneity within the theme and external homogeneity among the different themes identified were assessed using a dual criterion judging category (31). Homogeneity was assessed by reading through the transcribed data coded for a specific theme. This was done to ensure coherence among all the data represented within a theme. Candidate themes that were not coherent were recoded, reorganized, and sorted again, causing new themes to emerge or some to be merged with others. This also caused new subthemes to emerge in the study. The external homogeneity was assessed by reading through the entire dataset to ensure that each theme was a separate entity (though with some overlap) and that they together reflected the concept of the data set. Some of the identified themes had overlapping concepts in the “Abiye” program, so their points of overlap were reviewed and related to the objectives of the study. This allowed for more themes to emerge, and the codes in the miscellaneous theme were merged into appropriate places. The coding and recoding processes in this phase of the thematic analysis of this study continued until further coding and refinements did not yield any additional distinctive themes related to the objectives of the study. The themes devised in this phase are called “developed themes” (27).

##### Phase 5: Defining Themes

The developed themes were described by writing detail on what each theme captures in the data set. This was done to ensure that the details in the themes were within the scope of the objectives of this study. Appropriate subthemes were also developed for the themes where they were needed. The seven final themes that emerged from this thematic analysis were given appropriate titles that fully represent the details of their contents and how they fit into the study.

##### Phase 6: Report Writing

The findings that emerged from the analysis were shared with some of the participants to ensure the findings were credible and consistent with the perspectives of the study participants. This was to prevent the researchers' bias from influencing findings and the final report of the study. The themes with their subthemes that emerged in the thematic analysis of this study are shown in Table 3 and described in the Results section in detail and framed as it relates to context, mechanisms, and outcome to align with the study objectives. The contexts and mechanisms identified in this study and the description of the concepts were shared with the participants.

**Table 3:** Themes and sub-themes that emerged from the thematic analysis of the study.

**Themes**	**Sub-themes**
Financing Abiye safe motherhood program in Ondo State	Multiple funding models
	Coordination
	Incentives
Politics and Abiye safe motherhood program	Policy entrepreneur/actor power
	Sustainability
	Abiye design and idea
	Background maternal mortality issue
Human resources for health in Abiye safe motherhood program	Facility workers
	Health rangers
Facility and service delivery in Abiye safe motherhood program	Facility
	Service delivery
Data management in Abiye safe motherhood program	Tracking
	CEMDOS
	Monitoring and evaluation
Traditional birth attendant and Agbebiye program in Abiye safe motherhood program	TBA
	“Agbebiye” (safe midwives)
Community-related factors in Abiye safe motherhood program	Culture
	Community engagement
	Location

## Results

We structured our findings along with the three domains of realist evaluation: context, mechanism, and outcome, and formed CMO Configurations that guided the development of the middle-range theory.

### Context

We identified the roles of contextual factors in the “Abiye” safe motherhood program in Ondo State using a simplified framework (5). Thirteen contextual factors were identified under five principal areas. Cultural belief and traditional birth attendants both played inhibiting roles at some stages in the implementation of the “Abiye” program while the financial context inhibited the implementation of the program when the salary and incentives of the workers became irregular. All other contextual factors played enabling roles in the implementation of the program. Table 4 shows the roles of the contextual factors.

**Table 4:** Roles of contextual factors in “Abiye” safe motherhood program in Ondo State Nigeria.

**Principal factors**	**Contextual factors**	**Roles**	**Power, politics, policy interplay**
Situational	Poor background maternal health (Agenda setting event)	Enabling	Policy
	Political and policy entrepreneur	Enabling	Power, politics, and policy
Structural	Geographical	Enabling	Policy
	Health workforce	Enabling	Policy and power
	Health facilities	Enabling	Policy and power
Design	Financial	Enabling/inhibiting	Policy and power
	Community	Enabling	Policy and power
	Data management	Enabling	Policy
	Sustainability	Enabling	Policy and politics
Cultural	Cultural belief	Inhibiting	Policy
	Traditional birth attendant	Inhibiting	Policy
	Agbebiye program	Enabling	Policy and power
International community	International community	Enabling	Policy, politics and power

### Mechanisms

We determined mechanisms that facilitated or constrained the outcomes of the “Abiye” program. The mechanisms were grouped broadly into eight groups, each having a specific focus. Each of the mechanisms identified was justified based on the themes that emerged during the thematic analysis, and the relevance to the contextual factors which influenced the implementation of “the Abiye” program with the resulting outcomes. The lack of law for the sustainability of the “Abiye” program, demotivation due to irregular payment of salary and incentives, and poor health-seeking behavior, which was a mechanism within the community constrained the implementation of the program. Other identified mechanisms that facilitated the implementation of the program are policy entrepreneurship, commitment and will, legal nudge, central funding system, financial protection, knowledge acquisition, task shifting, health rangers' mechanisms, bonding, perceived trust, and good health-seeking behavior. Table 5 shows mechanisms that facilitated or constrained the program.

**Table 5:** Mechanisms that facilitated and constrained outcomes of the “Abiye” program.

**Mechanism (interplaying factors)**	**Facilitating**	**Constraining**
Political mechanisms—politics and Policy interplay	High-level policy entrepreneurship Commitment and Will that drove the program	No enabling law for a sustainability plan
	Nudge—Legal provisions that created CEMDOS and bill prohibiting TBAs from taking deliveries	
Funding mechanisms—policy and power interplay	Coordination by SPHCDB	
	Central funding system	
	Financial protection	Demotivation because of irregular payment of income
Human resource for health mechanisms—policy effect	Motivation and commitment by multiple incentives	
	Knowledge acquisition	
	Task shifting	
	Central pooling for human resources	
Health rangers mechanisms—policy effect	Bonding and perceived trust and support	
Facility and service delivery mechanisms—policy effect	Motivation	
	Service efficacy	
Data management mechanisms—policy effect	Accountability and data monitoring	
	Tracking	
Community mechanisms—power, politics and policy interplay	Mobilization and sensitization campaigns	Poor health seeking behavior
	WDC developmental activities	
	Good health-seeking behavior	
Traditional birth attendant/agbebiye—power, politics and policy interplay	Carrot and stick (Agbebiye)	

*WDC, Ward Development Committee*.

### Outcomes in the “Abiye” Safe Motherhood Program

#### Outcomes

##### Health System Strengthening

The “Abiye” safe motherhood program in Ondo State apart from achieving the set goals, also contributed to the strengthening of the Ondo State health system. It affected all the building blocks of the health system in the state. The health workforce, health financing, service delivery, information management system, medicine and technology, and leadership were all strengthened in the state during the implementation of the program. The supply and demand components of the IPT are related to the health system building blocks and this is specified in Table 6.

**Table 6:** Health system strengthening in Ondo State through the “Abiye” program.

**Health system building block**	**Achievements/effects**
Human resources for health	Recruiting of qualified health professionals into the state healthcare system
	Training and retraining of health professionals in relevant maternal health themes
Service delivery	Provision of BEOC and CEOC services at the appropriate health facilities
	Availability of 24 h services at the health facilities
Financing	Increase from 2.9% in 2009 to 11% in 2014 of the state budget to healthcare
	Multiple models of funding: PBF, DFF, NHIS and donor grants
Medicines and technologies	Availability of all drugs and materials at no cost to the women
	Equipping of the health facilities with equipment needed for lifesaving interventions
Leadership	Provided leadership for the development of other thematic health areas in the state.
Information	The HMIS in Ondo State was repositioned by the data management system of the “Abiye” safe motherhood program
	Tracking was central to the program and for generating data

*BEOC, Basic Emergency Obstetric Care; COC, Comprehensive Emergency Obstetric Care*.

Table 6 shows the strengthening of the health system in Ondo State through the “Abiye” program.

*As a key policymaker, I knew I had to do something to reduce maternal mortality. The short period I spent in clinical practice exposed me to harrowing experiences of maternal mortality which I have never forgotten. These experiences made me take the bull by the horns when I had the mantle of leadership. I decided to make a change in maternal mortality in Ondo State*.

**Key Informant 01**

*Recruiting health professionals of different cadres was a priority for the implementation of the “Abiye” program. We recruited CHEWs, nurses, midwives, and doctors for the program in the various facilities. We employed eight obstetricians and six pediatricians in the two MCH hospitals specifically built for the “Abiye” program. The number of consultants in the state workforce increased from 4 in 2007 to 72 by 2016*.

**Key Informant 01**

##### Skewed Prioritization and Funding

At the onset of the statewide implementation, the “Abiye” safe motherhood program in Ondo State was a priority of the state government. The political priority it generated in the state affected the focus and prioritization of some other programs within the health sector. Funding for the health sector was also skewed in favor of the “Abiye” program. The other non-“Abiye” facilities and non-maternal child health services were not well funded. This initial outcome was adjusted by the coordinating activities of the Ondo State Primary Health Care Development Board (SPHCDB), which ensured funds were appropriated to all units in primary health care in Ondo State.

*The “Abiye” committee coordinated how funding was done from the SMOH. We had a few sources of funds and had to strategize according to our priorities. We had some periods of skewed funding in favor of the “Abiye” program at the expense of other areas and in favor of designated “Abiye” facilities in the wards. The committee tried to achieve balanced funding during this period, but it was challenging*.

**Key Informant 04**

##### Community Engagement and Development

The “Abiye” program had a community engagement framework and power play in all the LGA which involved community participation in the program implementation and involved some community members in sensitization campaigns. The stakeholders' forum and Ward Health Development Committee (WHDC) formed during the “Abiye” program functioned for the development of the communities beyond just the “Abiye” program. For example, in the development of the sanitary system, water system, road and security networks in some of the communities.

##### Economic Empowerment and Development

The “Agbebiye” program (meaning “Safe Midwife” in the Yoruba language), a subsidiary of the “Abiye” safe motherhood program, had vocational skill training for Traditional Birth Attendants (TBAs) as part of the central plan of the policy when it was initiated. The TBAs were trained in a new specific vocational field for some time and were given start-up money for them to establish their new trade. They were economically empowered by this process, which led to some of them leaving the practice of delivering pregnant women. Furthermore, the recruitment of qualified health professionals like physicians, midwives, and nurses into the state as part of the policy of the intervention brought with it more socio-economic activities and investment opportunities into the state.

##### Increased State Health Wage Bill

The massive recruitment of health professionals into the state healthcare sector increased the health wage bill in the state resulting in an unintended outcome for the state government.

### CMO Configuration

#### Politics in CMO Configuration

A new government that prioritized reducing maternal mortality in Ondo State gave a window of opportunity for the implementation of the “Abiye” safe motherhood program. This favorable policy environment further ensured the enactment of the Confidential Enquiries in Maternal Deaths in Ondo State (CEMDOS) law in Ondo State. The commitment and will of the political actors (the State governor and the Commissioner of Health) to the program and the nudge by the legal system in the state facilitated the “Abiye” safe motherhood program that ensured the full implementation of the program. However, the lack of a definite legal sustainability plan for the “Abiye” program was a constraining mechanism that affected its implementation of the program.

*The data on maternal deaths in the state was an embarrassment to South-West Nigeria. This drove us to action. We also knew from different pieces of evidence that these deaths were highly preventable if we did the right things in our setting, and that is what we did*.

**Key Informant 02**

*We actually had plans to sustain “Abiye” in the state through the social protection bill but it never worked out. Also, the residential card, (“Kaadi Igbeayo”) we implemented had some challenges which affected sustainability funding plans for the “Abiye” program*.

**sKey Informant 01**

*The burden of neighboring state patients at the later stage of the program made us include “kaadi Igbeayo” (residency card) as a requirement to access free maternal health. We treated every pregnant woman but discharged them after presenting the card or full payment*.

**Key Informant 03**

#### Human Resources for Health in CMO Configuration

The recruitment of qualified health professionals, training and retraining of in-service staff, initiating the Health Ranger scheme, and multiple incentives for the specific service delivery rendered by the health care workers were the context of the program. The motivation from the multiple incentives was a mechanism that facilitated the implementation of the program making the workers always available and ready to ensure the pregnant women were delivered safely. Continuous improvement and development of the knowledge and skills of the health workers was another facilitating mechanism in Human Resources for Health in the implementation of the “Abiye” program. Another mechanism was the task shifting process during the “Abiye” safe motherhood program which was initially resisted, but when it was accepted and implemented, ensured more skilled attendants were at the delivery of pregnant women in the state. The state also used the mechanism of the pooling system for human resources for health within the state when the midwives of the Midwives Service Scheme (MSS) from the Federal government were integrated into the “Abiye” program.

*Health Rangers (CHEWs) were a game-changer in “Abiye” program. They were recruited and were tasked with ensuring safe delivery for all pregnant women. We did a task-shifting process to equip them to be skilled attendants proficient at managing pregnancies, but this became a battleground for some time with our midwives and nurses. We had 5 midwives and 23 nurses in a local government with over 10,000 pregnant women. There was an obvious need for task shifting and equipping of these CHEWs to help reduce maternal deaths*.

**Key Informant 02**

#### Funding in CMO Configuration

The “Abiye” safe motherhood program was implemented in the context of multiple sources of funding, different funding models (state government funding, performance-based financing, donor funds, and decentralized facility funding), and financial risk protection for pregnant women and their children. This removal of financial risk facilitated facility delivery among the women. The central funding system mechanism was an interplay of power and policy which facilitated the implementation and ensured effective use of resources in the state to achieve their outcomes.

*We partly funded the program from the state fund. This showed development partners our commitment and sincerity of purpose to reduce maternal mortality within the state. We had limited funds available for this but we consistently increased our total health financing from 2.9% of the state budget in 2009 to 11% by 2014*.

**Key Informant 01**

*State funding declined during the national economic recession period. This affected our implementation during this period, and state full ownership of the program*.

**Key Informant 04**

#### Facility and Service Delivery in CMO Configuration

The renovation, equipping, and building of facilities specifically for the “Abiye” program motivated health workers to be committed and ensured availability of needed life-saving interventions and effectiveness in service delivery The referral ambulance service of the program ensured pregnant women requiring lifesaving interventions get to the health facilities immediately, while the use of Toll-free Closed User Group (CUG) in the “Abiye” program by the different participants ensured utilization of the program and thereby facilitated its implementation.

#### Data Management in CMO Configuration

Accountability and monitoring in the “Abiye” program were mechanisms that facilitated the outcome achieved because of regular data collection, monitoring, and review of the activities of the health rangers in the communities. The tracking system in the program by the State Primary Health Care Development Board and performance-based financing, which required data to measure performance, also facilitated its outcome.

*A word that describes “Abiye” is tracking. Tracking of pregnant women in the communities, keep tracking them during their period of pregnancy and track them in labor to delivery, and keep track of them to the postnatal period*.

**Key Informant 07**

*PBF (performance-based financing) is very good. It helped our facility to grow and render better maternal services. We employed temporary staff, including a medical doctor through it, which helped improve the utilization of our services. We did not have to go through the bureaucracy of the SPHCDB in Akure (the state capital) to make decisions on some funding and recruitment issues*.

**IDI 01**

*PBF is a strict funding system. Our funders are results-driven. We had to put in our best to achieve these results or we might lose our funding. We also needed to collect accurate data like phone numbers, house addresses (description to the house), and names (called at home), which we did not routinely do formerly. Falsified or unverifiable data is not accepted and I know some facilities lost their funding because of this*.

**IDI 02**

#### Community in CMO Configuration

**Figure 3:**
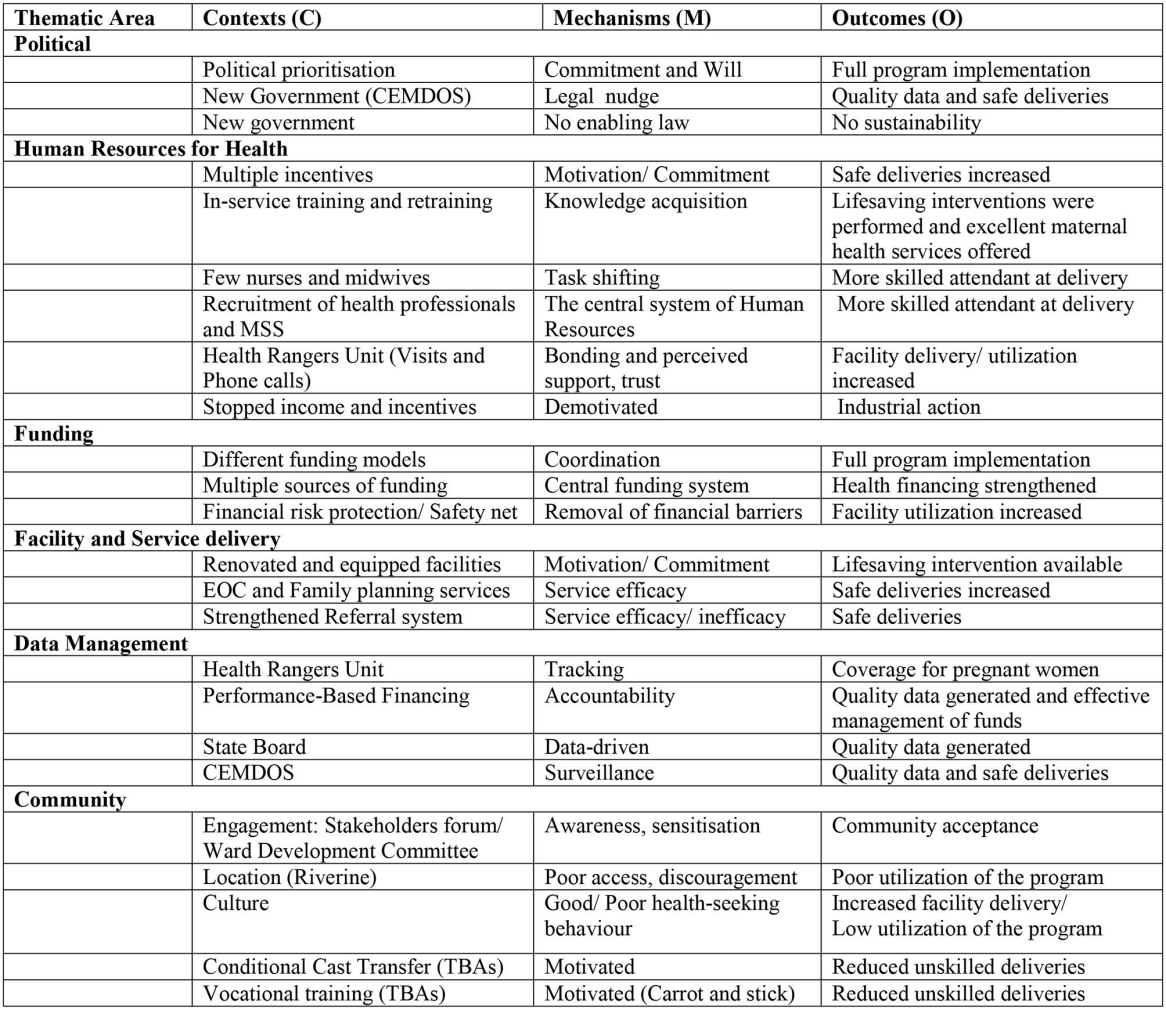
Context, mechanism, and outcome configuration in Abiye safe motherhood program.

The mobilization campaign in the communities was a mechanism that facilitated the implementation of the program in the state. The community members and engaged before the implementation started and were involved in the decision-making process at the community level through the activities of the WHDC, which was another mechanism at the community level. The TBAs within the communities were also motivated by incentives to bring pregnant women to the health facilities to deliver thereby further reducing maternal mortality. The “carrot and stick” approach was used for the TBAs in the communities initiated by the Agbebiye program. This facilitated the outcome of the “Abiye” program in Ondo State. Discouragement of some community members because of the bad road terrain in their communities was a constraining mechanism that affected the implementation of the “Abiye” safe motherhood program in such communities. See Figure 3 for CMO Configurations of the study.

### New Program Theory

“Provision of sustainable free quality maternal care to all pregnant women by motivated health professionals within a comprehensive health design in fully engaged communities, with a background of strong political will and prioritization, would improve utilization of the health facilities and skilled delivery thereby sustainably reducing maternal mortality in Ondo State.”

**Figure 4:**
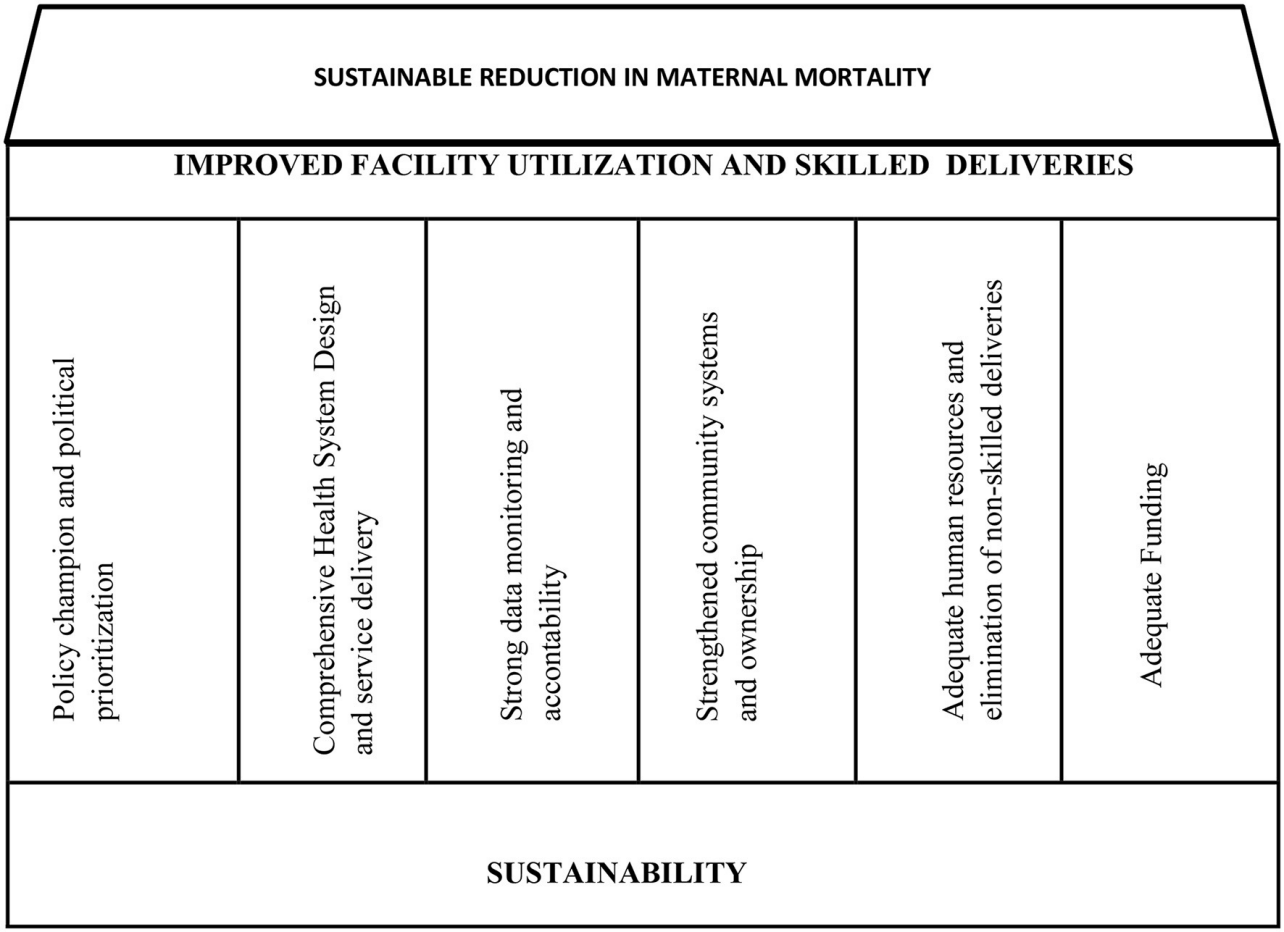
Framework for maternal mortality reduction programs.

Based on this new program theory, a Framework for maternal mortality reduction program was developed, which can be adopted in LMIC where maternal mortality is a challenge (Figure 4).

## Discussion

The gap between policymaking and evidence generated from studies in LMIC is a challenge. Multiple evaluations of intervention programs have focused on the effectiveness and not on “what works for whom, how, why and under what circumstances”. The realist approach, which we applied in this study, explicitly explored the implementation of the “Abiye” safe motherhood program in Ondo State identifying the roles of contextual factors, the effects of mechanisms that triggered various outcomes. Furthermore, we unveiled the “black-box” of program implementation and Health System Strengthening.

The contextual factors of the implementation of the “Abiye” safe motherhood program in Ondo State that emerged in this study are situational factors (agenda-setting event, political and policy entrepreneur), structural factors (geographical, health workforce, and health facilities), design factors (financial, community, data management, and sustainability), cultural factors (cultural belief, traditional birth attendant, “Agbebiye” program), and international community factor (International community). Most of these factors with their context have been described as contextual factors in previous realist evaluation studies. Marchal et al. (10) focused on hospital management and performance in Ghana, as well as reported health workforce, data management, and health facilities structure as found in this study were contextual factors that influence hospital management and performance in their setting. Similarly, Marchal et al. reported that in Morocco political entrepreneurs, community and finance were contextual factors that influenced fee exemption policies (34). Other realist studies which have spanned over a decade in low, middle, and high-income countries (8, 9, 35, 36) reveal agenda-setting events, geographical, cultural, and international community as contextual factors that influence program implementation as found in this study. These factors have been recurring for over a decade and have been reported across various economic settings. They are therefore important factors to be considered to ensure the success of any program irrespective of the socioeconomic pedigree.

However, the “Agbebiye”, sustainability, and financial context reported in this study have not been reported in previous realist evaluation studies. This might be because the “Agbebiye” program was specific to the “Abiye” safe motherhood program, and the sustainability context reported in this study was also specific to the program. Nonetheless, we propose that for similar safe motherhood projects, program implementers could adapt the concept of Agbebiye into their setting, as this study shows it to be a program enabler.

The financial context reported in this study is different from the previously reported financial context in realist studies because of the multiple funding models, central funding system, and coordination system of the financial context in the “Abiye” program. The multiple sources of funds coupled with good coordination probably contributed to the good outcomes of the Abiye safe motherhood program.

In this current study, several facilitating and constraining mechanisms were identified to have affected the Abiye safe motherhood program implementation/outcomes. As found in this study, motivation and knowledge acquisition by health workers as program facilitating mechanisms has been previously reported by a prior realist evaluation study in Nigeria on the use of neonatal and pediatric pulse oximeters in 12 hospitals (37). Similarly, Marchal et al. (10) in Ghana and Makumbang et al. (35) in South Africa also reported these mechanisms as program implementation/outcome facilitators. Also, bonding and perceived support, trust, removal of financial barriers, commitment, and service efficacy as found in this study were previously reported by Makumbang et al. to be facilitating mechanisms for successful program implementation. These mechanisms should thus be given careful consideration and properly woven into the planning and implementation of the subsequent health-related program as they have been shown to facilitate program success.

The policy entrepreneur's commitment and nudge by legal provisions were facilitating mechanisms identified in the political theme that emerged in this study. Similar facilitating mechanisms have been reported in a London realist evaluation study on modernizing health service (8). The awareness, engagement, and good health-seeking behavior were the facilitating mechanisms identified in the community theme that emerged in this study, which are similar to findings in a realist evaluation of Community Health Committees in Nigeria (38) which identified community connectors as a facilitating mechanism. Another realist evaluation in Bangladesh (9) on maternal and newborn health programming identified participating in health facility activities as a facilitating mechanism for the community women. The facilitating mechanisms identified in the data management theme of this study are accountability and data monitoring which are similar to some of the facilitating mechanisms in the realist evaluation study in Ghana (10) and Bangladesh (9).

There are three facilitating mechanisms identified in this study that were not reported in previous realist evaluation studies to the best of the authors' knowledge. These are the task-shifting mechanism, central funding system mechanism, and tracking mechanism. However, they have been identified as forms of causal/ actor process mechanisms (39–41) which facilitate program implementation as found in this study.

The constraining mechanisms identified in this study were demotivation of the health workers when program funds dwindled and the health workers' payments were adversely affected, and poor health-seeking behavior which was a cultural factor in certain riverine communities with poor road access. Demotivation and poor health-seeking behavior have been reported as constraining mechanisms in some realist evaluation studies in Nigeria (37), Ghana (10), South Africa (35), Bangladesh (9), and London (8). Hence, more attention should be paid to the vulnerable populations and ways to improve their health-seeking behavior to access lifesaving interventions at the planning and implementation stages of such programs.

The lack of legal nudge for sustainability identified in this study as a constraining mechanism in this study was not reported in previous realist evaluation studies. This is possibly why the successful implementation of some programs starts to dwindle with time as found in this study. It is thus imperative that legal nudges for sustainability and identified and incorporated at the planning stage of intervention programs. Limited literature exists on the interplay of power, politics, and policy in the health system (42, 43). Institutionalization has been described as a vehicle for the interplay of these three concepts (44), which was also evident in this study. The interplay of these concepts facilitated the provision of a safety net in this study which is similar to existing knowledge on the effects of these concepts on health equity, though the evidence varies in different studies (45, 46). The influence of the international community was similar to documented evidence from Millennium Development Goals (MDG) implementation (47, 48) and funding in developing countries (49, 50).

The framework for maternal mortality reduction programs developed in this study could be applied in different settings. This framework is a guide around which maternal mortality reduction programs and also other public health interventions can be built. The key elements of this framework are: (a) policy championing and political prioritization, (b) comprehensive health system design, (c) strong data monitoring, (d) strengthened community systems and ownership, (e) elimination of non-skilled deliveries, and (f) adequate funding.

Policy championing and sustainable political prioritization in maternal health programs have been reported in previous studies in Nigeria and sub-Saharan Africa (51). Similar to this, policy championing and political prioritization have also been reported as vital in other public health intervention (52) and reported in multiple studies as being central to the achievement of Universal Health Coverage in any society (53, 54). A comprehensive health system design that is sustainable is necessary for maternal programs. It comprises the health system and the building blocks which have been reported as needed for development in healthcare services (55). The community's engagement and participation in maternal health programs have been reported to promote the acceptability and sustainability of such programs (56). Public health intervention programs have also been reported strengthened by community ownership (57). Elimination of non-skilled deliveries was also a component of the framework developed in this study. Some studies in Nigeria (58) and Ethiopia (59) have reported collaboration with TBAs while the National Reproductive Health Policy 2017 has eliminated non-skilled deliveries as a strategy for reducing maternal mortality in Nigeria (60). However, phasing out non-skilled deliveries might be a challenge, but a well-designed maternal health program would reduce their effect and may make them extinct gradually. Sustainable adequate funding is important in maternal mortality reduction programs and health system strengthening (61) as shown in this study. To sustain the free maternal health services provided from antenatal to the postnatal period, contributory Community Based Health Insurance (62) in which every adult pays a token annually and the government subsidizes it or the Social Health Insurance system (63) has been reported in the literature. In Nigeria, the “Investment Case” 2017–2030 of the Federal Ministry of Health for reproductive health, maternal, newborn, child, adolescent health and nutrition (64) also highlighted the need for adequate funding of maternal health programs and has also been reported in some studies in sub-Saharan (65) Africa and globally (66).

This study helps in filling some of the research gaps in the realist evaluation approach. It used a realist approach to evaluate the implementation and possible outcome of a safe motherhood initiative in a low-middle income. It has brought to the fore how specific contextual factors, their mechanisms, and outcomes influence program implementation. In addition, a framework for maternal mortality reduction programs was developed which could be applied in different settings, and also for other public health intervention programs. Thus, findings from this study are useful for government at all levels, policymakers, and program managers in public health programming in Sub-Saharan Africa and other LMICs.

### Limitations of the Study

The realist evaluation approach of the study did not allow for any causal relationship/ inferences to be made but it showed the different factors (context and mechanisms) that potentially influenced the outcome of the program. Analyzing a public policy intervention program like the “Abiye” safe motherhood program is prone to social desirability bias. However, a detailed explanation of the objectives of the study to the participants, highlighting the possible benefits to Ondo State and reassurance of confidentiality is believed to have sufficiently encouraged the participants to give valid responses. Thematic analysis was used for qualitative analysis, and it is prone to the researchers' subjective views but this was addressed by reviewing every phase of the thematic analysis process with the recorded audiotape of the data set, thereby ensuring all the detail in the themes that emerged represent the data collected in the study.

## Conclusion

Realist evaluation is an iterative process that looks beyond the surface to generate evidence. By applying the realist approach, we generated pieces of evidence that can be adapted for policymaking in public health interventions in LMIC, especially for maternal mortality reduction programs.

## Data Availability Statement

The raw data supporting the conclusions of this article will be made available by the authors, without undue reservation.

## Ethics Statement

The studies involving human participants were reviewed and approved by Health Research and Ethics Committee of the Institute of Public Health, Obafemi Awolowo University, Ile-Ife, Osun State and its HREC Number is IPHOAU/12/830; Health Research and Ethics Committee of Ondo State Ministry of Health and its HREC Number is OSHREC/25/09/2017/019. The patients/participants provided their written informed consent to participate in this study.

## Author Contributions

OO developed the concept of the study, co-designed the proposal and methodology, analyzed the data, interpreted the data, drafted the article, and approved the final version. AF reviewed the concept of the study, co-designed the proposal and methodology, reviewed and interpreted data, repeatedly reviewed the draft article, and approved the final version. All authors contributed to the article and approved the submitted version.

## Conflict of Interest

The authors declare that the research was conducted in the absence of any commercial or financial relationships that could be construed as a potential conflict of interest.

## Publisher's Note

All claims expressed in this article are solely those of the authors and do not necessarily represent those of their affiliated organizations, or those of the publisher, the editors and the reviewers. Any product that may be evaluated in this article, or claim that may be made by its manufacturer, is not guaranteed or endorsed by the publisher.
